# Subcortical volumes are reduced in short-term and long-term abstinent alcoholics but not those with a comorbid stimulant disorder^☆^

**DOI:** 10.1016/j.nicl.2013.06.018

**Published:** 2013-07-11

**Authors:** George Fein, David Fein

**Affiliations:** a Neurobehavioral Research, Inc., 1585 Kapiolani Blvd., Ste. 1030, Honolulu, HI 96814, USA; b Department of Psychology, University of Hawai'i, 2530 Dole Street, Sakamaki C 400, Honolulu, HI 96822, USA

**Keywords:** Alcoholism, Subcortical

## Abstract

Chronic alcohol abuse affects brain structure and function. We examined subcortical structure volumes in 77 short (6–15 week) and 90 long (multi-year) term abstinent alcoholics, along with 74 controls. We used a 3T Siemens MPRAGE sequence for image acquisition and FSL FIRST software for measuring subcortical volumes. When examining alcoholics without a comorbid stimulant disorder we found reduced hippocampal, pallidum and thalamus volumes in short term abstinence compared to a non-substance abusing control sample with numerically smaller yet still significant reductions compared to controls in long term abstinence. When examining alcoholics with a comorbid stimulant disorder, no difference from controls was found for any subcortical volume. Alcoholics with a stimulant disorder had significantly larger subcortical volumes than alcoholics without a stimulant disorder.

This study replicates past research showing that chronic alcohol abuse is associated with lower subcortical volumes in short-term abstinent chronic alcoholics and extends this finding, although with smaller effects to long-term abstinent samples. The absence of this effect in the presence of a comorbid stimulant disorder suggests either a protective effect of stimulant abuse/dependence or that the measurements reflect the aggregate of alcohol dependence associated atrophy and stimulant abuse associated inflammation. Associations with function suggest the second of these two alternatives.

## Highlights

• Subcortical atrophy
• Chronic alcohol dependence
• Comorbid stimulant disorder

## 1 Introduction

Substantial changes in brain morphology mark the stages of alcoholism, including hazardous drinking, dependence, recovery and relapse (Cardenas et al., 2007; Rohlfing et al., 2006). Magnetic resonance imaging (MRI) has been extensively used to study morphological changes in subcortical structures that are associated with alcohol use disorders (AUD) (Cardenas et al., 2011). Animal experiments (Bonthius et al., 2001), human postmortem brain studies (Harding et al., 1997), and human imaging studies (Agartz et al., 1999; Sullivan et al., 1995, 1996) all show that chronic heavy alcohol consumption damages the hippocampus; additionally, changes in hippocampus-related functions such as visuospatial learning and memory (Berthoz, 1997; Ghaem et al., 1997; Hartley et al., 2007; Iaria et al., 2003; Lavenex et al., 2006; O'Keefe, 1990; Santin et al., 2000) are among the most consistently found consequences of chronic alcoholism in humans and in rodents model (Beatty et al., 1996; Bowden, 1988; Corral-Varela and Cadaveira, 2002; De Renzi et al., 1984; Matthews and Morrow, 2000; Nixon et al., 1987; Oscar-Berman and Ellis, 1987; Riege, 1987; Shelton et al., 1984). Damaging effects on the hippocampus are believed to result from the combined effects of ethanol-induced glucocorticoid elevation, compromised nutrition, and oxidative stress. In previous research on long-term abstinent alcoholics (LTAA) in our laboratory, (Sameti et al., 2011) we did not find reduced subcortical (including hippocampal) volumes in LTAA, but did find significantly lower hippocampus (and amygdala) volumes in multi-year abstinent alcoholics with co-morbid psychiatric disorders.

Research has also focused on AUD disruption of brain reward pathways (Koob, 1999; Koob and Kreek, 2007; Kreek and Koob, 1998; Makris et al., 2008; Wise, 1998), including prefrontal cortex and the subcortical striatopallidal and extended amygdala systems. Repeated chronic abuse and withdrawal of alcohol and drugs can change one's hedonic set point (e.g., raising thresholds), increasing substance dependence. Alcoholics have shown smaller volumes of reward-related structures (nucleus accumbens and amygdala, along with dorsolateral prefrontal cortex and anterior insula) compared to non-alcoholic controls (Makris et al., 2008), with these smaller volumes associated with impaired memory.

A hallmark of alcohol abuse is poor decision making with regard to alcohol use. The Iowa Gambling Task (IGT) (Bechara et al., 1994), simulates real-life decisions with uncertain rewards and punishments. Impaired performance on the IGT has been interpreted in terms of an inability to attach appropriate negative emotional valence to negative consequences. Currently active or recently detoxified alcoholics have demonstrated impaired performance on the SGT (Bechara and Damasio, 2002; Bechara et al., 2001; Mazas et al., 2000), and we (Fein et al., 2004a) have shown persistent impaired performance on the SGT in LTAA. We (Fein et al., 2006) found that multi-year abstinent alcoholics who were impaired on the SGT had bilaterally smaller amygdala volumes compared to controls.

There is a body of research observing hypertrophy in basal subcortical structures in stimulant abusing individuals. Chang et al. (2005) found larger putamen and globus pallidus in 50 METH users vs. controls. In that study, METH users had normal cognitive function, and those with smaller striatal structures had poorer cognitive performance and greater cumulative METH usage. Jernigan and Gamst (2005) examined the separate and combined effects of methamphetamine dependence and HIV infection on brain morphology. They found that methamphetamine dependence was associated with basal ganglia and parietal cortex volume increases, with neurocognitive impairment associated with the methamphetamine dependence volume increases (a different pattern of associations from the Chang paper). However, not all studies of METH found enlarged subcortical volumes (see Chang et al.'s, 2007 review). Thompson et al. (2004) reported reduced hippocampal volumes in chronic METH users. More recently, Ersche et al. (2011) found that cocaine users had increased gray matter volumes in the caudate nucleus, and the ventral striatum. Ersche et al. (2012) also reported gray matter medial-temporal lobe and basal ganglia volume increases in both stimulant-dependent individuals and their non-drug abusing siblings.

The purpose of the current study was to examine subcortical volumes in short-term and long-term abstinent pure alcoholics and alcoholics with co-occurring stimulant abuse or dependence. We hypothesized that we would see lower subcortical volumes in short-term abstinent alcoholics without comorbid stimulant dependence, but not in those with comorbid stimulant dependence. We hypothesized that such individuals would have alcohol dependence induced subcortical atrophy masked by stimulant dependence induced subcortical inflammation. We did not expect to see smaller subcortical volumes in long-term abstinent individuals with alcohol dependence only, but might see smaller hippocampal and amygdala volumes in such long-term abstinent with alcohol dependence only if they also had comorbid internalizing psychiatric disorders (as predicted from our prior study of long-term abstinent alcoholics).

## 2 Methods and materials

### 2.1 Participants

Short-term (6–15 week) abstinent alcoholics (STAA, n = 77), LTAA (greater than 18 months abstinent, n = 90) and non-substance abusing controls (NSAC, n = 74) were recruited from the island of Oahu (Table 1). Abstinent alcoholics were recruited through advertisements and fliers posted in various treatment programs and 12-step recovery meetings, and met DSM-IV-R lifetime criteria for alcohol dependence (American Psychiatric Association, 1994). Forty-one STAA and 43 LTAA also met lifetime criteria for stimulant (cocaine and/or methamphetamine) abuse or dependence. For STAA, 12 had a cocaine disorder, 18 had a methamphetamine disorder, and 10 had both cocaine and methamphetamine disorders. For LTAA, 19 had a cocaine disorder, 9 had a methamphetamine disorder, and 15 had both cocaine and methamphetamine disorders. Subjects' substance use history was gathered using the Lifetime Drinking History instrument with timeline follow-back methodology (Skinner and Sheu, 1982), administered separately for alcohol and for each other substance used. In addition, subjects completed the computerized Diagnostic Interview Schedule (C-DIS) (Levitan et al., 1991) to ascertain externalizing, anxiety or mood disorder diagnoses and symptom counts.

A breathalyzer test to screen for alcohol (Alco-Sensor IV, Intoximeters, Inc., Saint Louis, MO) and a saliva screen for drugs (Oral Fluid Drug Screen Device, Innovacon, Inc., San Diego, CA) was performed for all subjects on each testing day, with negative findings required for participation (no subjects failed screens). Participants received monetary compensation for their participation. Exclusion criteria for all groups included: a) significant history of head trauma or cranial surgery; b) current or lifetime history of diabetes, stroke, or hypertension that required medical intervention; c) current or lifetime history of a significant neurological disorder; d) clinical or laboratory evidence of active hepatic disease; e) clinical evidence for Wernicke–Korsakoff syndrome, and f) lifetime diagnosis of schizophrenia or schizophreniform disorder (assessed by the C-DIS). For this current study, subjects with lifetime dependence on opiates or marijuana were excluded.

### 2.2 Imaging data acquisition and analysis

MRIs were collected on a 3.0T Siemens Trio Tim platform, located at the Queens Medical Center in Honolulu, HI. For each subject, we acquired a sagittal T1-weighted (MPRAGE) image (TR = 2200 ms, TE = 4.1 ms, TI = 1000 ms, acquisition matrix = 256 × 256) with 160 slices at 1.0 mm thickness and a Fluid Attenuated Inversion Recovery (FLAIR) image (TR = 9100 ms, TE = 83 ms, TI = 2500 ms, acquisition matrix = 204 × 230) with 44 slices at 3 mm thickness. A neuroradiologist read all MRI scans. All scans were free from abnormalities other than white matter signal hyperintensities (WMSH) visualized on the FLAIR images. Subcortical hyperintensities were identified on only a handful of subjects in the current study samples.

We measured subcortical brain structures using FSL's FIRST software (FMRIB Image Registration and Segmentation Tool) (Patenaude, 2007), a method that has been used by us and others to successfully measure subcortical volumes in a number of recent investigation (Angstrom et al., 2004; Corthorn et al., 1997; Figueroa Cave, 1997; Goodro et al., 2012; Markov et al., 1997; Rinehart et al., 1997; Sameti et al., 2011). The following structures were extracted and their volumes measured for all T1-weighted MR images: left and right thalamus, caudate, putamen, pallidum, hippocampus, amygdala, and nucleus accumbens. When boundaries of the delineated structures were visually inspected, we observed boundary underestimation of lateral ventricles (and corresponding overestimation of subcortical structures bordering on the lateral ventricles) in MRIs of subjects with ventricles significantly larger than those in the MNI152 standard template. To ensure a more accurate segmentation, MRIs were registered MNI152 standard space template using FSL's FNIRT (FMRIB's Nonlinear Image Registration Tool). The warped (registered) MRIs were then processed through FIRST to extract the surface mesh of each of the subcortical structures. The surfaces were then transformed back to the original MRI space, filled and boundary corrected using the FSL tool “first_utils” (preventing voxel overlap between structures). Boundaries of each structure were visually inspected for gross errors. Visual inspection also showed that subcortical volume estimates were unaffected by subcortical hyperintensities, when present. Each extracted structure was measured in cubic millimeters. Cranium size estimation (an estimate of premorbid brain size) was also performed using FSL's SIENAX (Structural Image Evaluation, using Normalization, of Atrophy) tool. We have previously shown that the FSL cranium size index is an excellent surrogate for the intracranial vault volume (Fein et al., 2004b).

### 2.3 Cognitive assessment

The Cambridge Automated Neuropsychological Test Battery (CANTAB, Cambridge Cognition Ltd.) was used for the cognitive assessment. The following tests were administered to all participants: Affective Go/No-go, Big/Little Circle, Delayed Matching to Sample, Intra-Extra Dimensional Shift, Spatial Recognition Memory, Spatial Working Memory, Motor Screening, Matching to Sample, and Reaction Time.

### 2.4 Statistical analysis

The General Linear Model (GLM) (SPSS Inc., 2009) was first used to determine whether subcortical volumes were correlated with the cranium size index. Since all subcortical volumes were significantly positively correlated with the cranium size, we used linear regression to adjust each structure volume for the cranium size index. For each subcortical structure, we then added the left and right volumes, resulting in 7 cranium size adjusted volumes per subject. We compared NSAC to STAA DOA (dependent on alcohol only), STAA DASD (dependent on alcohol with a co-occurring stimulant disorder), and to both LTAA DAO and LTAA DASD. We also compared STAA DAO with STAA DASD and LTAA DAO with LTAA DASD. For each of these analyses, we first performed a multivariate analysis across the seven subcortical volumes. Control for multiple comparisons was accomplished using a variant of Fisher's protected *t*-test, where comparisons of individual structures were only examined if the multivariate test across structures was significant. Partial eta squared was used as the measure of effect size. Partial eta squared measures the percent of variance of the dependent variable that is independently accounted for by the independent variable.

We examined the associations (separately for STAA DAO, STAA DASD, LTAA DAO, and LTAA DASD) between subcortical volumes and measures of alcohol, stimulant, and nicotine use (including days abstinent), and with mood and anxiety diagnoses using Spearman correlations. We also examined associations with subcortical volumes of CANTAB tests that showed impairments in STAA vs. NSAC (Affective Go/No-go, Delayed Matching to Sample, Motor Screening, and both Spatial Recognition Memory and Spatial Working Memory), and compared STAA DAO with STAA DASD on those CANTAB tests.

## 3 Results

### 3.1 Demographics

Table 1 presents the demographics for the study. The study groups and (within STAA and LTAA) DAO and DASD were comparable on age and did not differ on any alcohol use measure nor on the prevalence of psychiatric comorbidities (Fein, in press). DAO and DASD differed on age of alcohol dependence diagnosis, with individuals with alcohol dependent individuals with a lifetime stimulant disorder meeting criteria for alcohol dependence 5–7 years earlier than those without a lifetime stimulant disorder (F_1,71_ = 16.5, p < .0001, es = 18.8% for STAA and F_1,86_ = 10.6, p = .002, es = 11.0% for LTAA).

### 3.2 Subcortical volumes

**Fig. 1:**
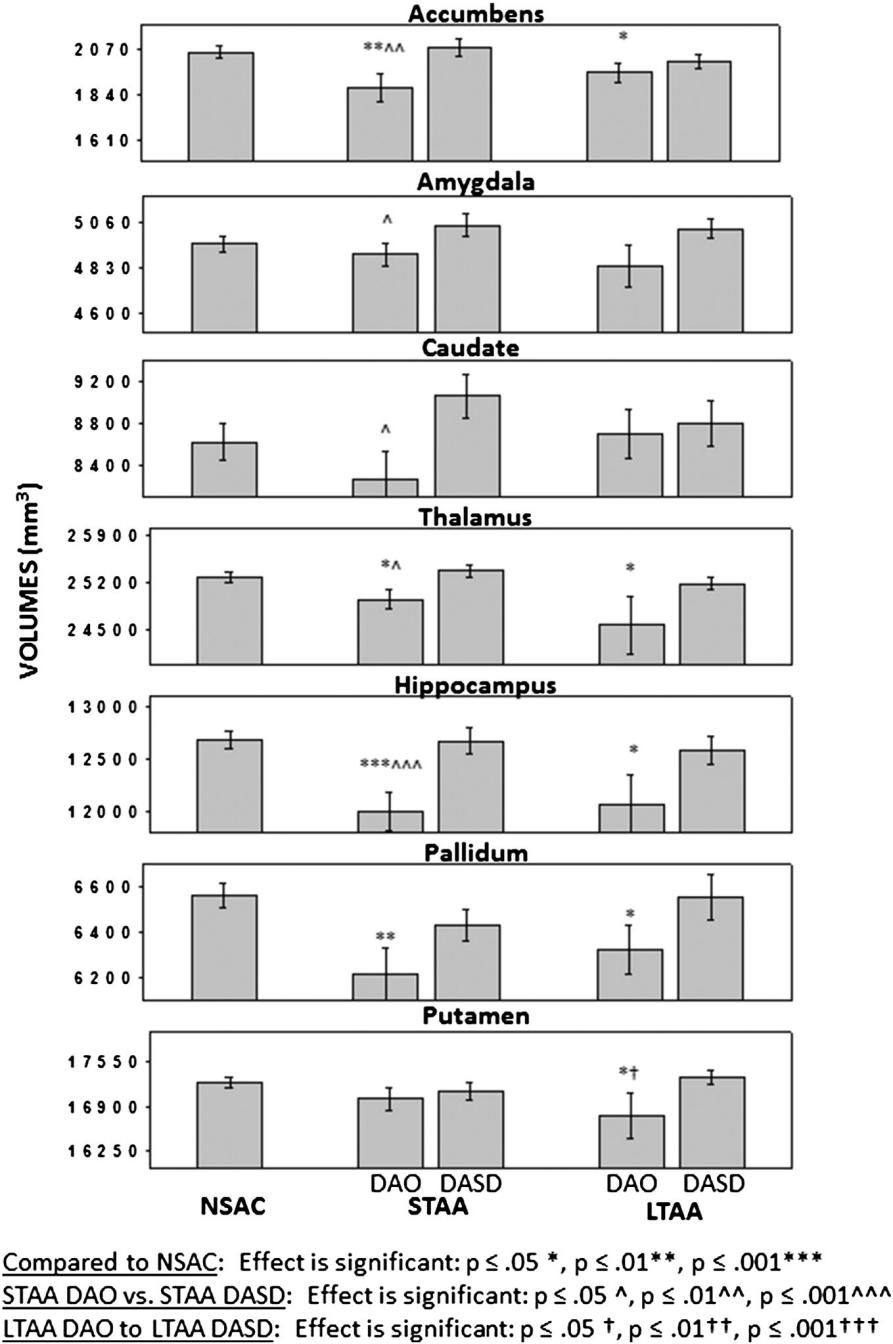
Volumes and effect sizes of the seven subcortical structures for the various subject groups. Abbreviations: LTAA, Long-Term Abstinent Alcoholics; NSAC, NonSubstance Abusing Controls; STAA, Short-Term Abstinent Alcoholics; DAO, Dependent on Alcohol Only; DASD, Dependent on Alcohol with a Stimulant Disorder (abuse or dependence).

Table 2 and Fig. 1 present the subcortical volume data.

STAA DAO vs. NSAC: Comparing NSAC and STAA DAO, the multivariate test was highly significant (Wilks' λ_7,100_ = 0.79, p < .001), with smaller volumes in STAA DAO for the accumbens (F_1,106_ = 8.95, p < .003, es = 7.8), hippocampus (F_1,106_ = 16.58, p = .001, es = 13.5), and pallidum (F_1,106_ = 9.10, P = .003, es = 7.0), and slightly larger volumes for the thalamus(F_1,106_ = 3.94, p = .05, es = 3.6). Including age as a covariate did not change the results.

STAA DASD vs. NSAC: Comparing NSAC and STAA DASD, the multivariate test was not significant (Wilks' λ_7,105_ = 0.91, p > 0.20).

STAA DAO vs. STAA DASD: Comparing STAA DAO and STAA DASD, the multivariate test was significant (Wilks' λ_7,67_ = 0.81, p = .047), with larger volumes in STAA DASD for the accumbens (F_1,73_ = 7.06, p = .010, es = 8.8), amygdala (F_1,73_ = 4.85, p = .031, es = 6.2), caudate (F_1,73_ = 6.15. p = .015, es = 7.8), hippocampus (F_1,73_ = 11.23, p < .001, es = 13.3), and thalamus(F_,1.73_ = 5.28, p = .024, es = 6.7). Including age as a covariate did not change the results.

LTAA DAO vs. NSAC: Comparing NSAC and LTAA DAO, the multivariate test was marginally significant (Wilks' λ_7,111_ = 0.887, p = .059), with smaller volumes in LTAA DAO for the accumbens (F_1,117_ = 4.17. p = .043, es = 3.4), hippocampus (F_1,117_ = 6.41, p = .013, es = 5.2), pallidum (F_1,117_ = 5.08, p = .026, es = 4.2), putamen (F_1,117_ = 4.92, p = .028, es = 4.0),and thalamus(F_1,117_ = 4.90, p = .029, es = 4.06). Including age as a covariate did not change the results.

LTAA DASD vs. NSAC: Comparing NSAC and LTAA DASD, the multivariate test was not significant (Wilks' λ_7,107_ = 0.927, p > 0.30).

LTAA DAO vs. LTAA DASD: Comparing LTAA DAO and LTAA DASD, the multivariate test was not significant (Wilks' λ_7,80_ = 0.937, p > 0.60).

STAA DAO vs. LTAA DAO: Comparing STAA DAO and LTAA DAO, the multivariate test was not significant (Wilks' λ_7,73_ = 0.872, p > 0.17). The same was true comparing STAA DASD and LTAA DASD (Wilks' λ_7,74_ = 0.940, p > 0.68).

Cocaine vs. methamphetamine disorders: Within STAA and LTAA, we compared subcortical volumes between subjects with a cocaine disorder to those with a methamphetamine disorder, excluding individuals with both cocaine and methamphetamine disorder. For STAA, there were no differences between those with the two types of disorder (Wilks' λ_7,23_ = 0.715, p > 0.29). For LTAA, there was a trend toward a significant difference between those with the two types of disorder (Wilks' λ_7,20_ = 0.548, p = 0.063), with lower volumes for cocaine disorder vs. methamphetamine disorder individuals for the accumbens, hippocampus and thalamus (all p's < 0.05).

### 3.3 Association of subcortical volumes with substance use and psychiatric diagnoses

For alcohol, cocaine and methamphetamine, lifetime and peak use and dose are highly intercorrelated (all r's > 0.85). Lifetime and peak use measures for cocaine and alcohol are also significantly correlated (r = 0.254 and r = 0.216, both p's < 0.05). Methamphetamine use measures were not correlated with either alcohol or cocaine use measures (all |r| < 0.13, all p's > 0.22). In STAA DAO, accumbens, caudate, and thalamus volumes were negatively associated with alcohol use (r's between − 0.328 and − 0.376, p's < .050, uncorrected for multiple comparisons). There was no significant subcortical volume by alcohol use associations in LTAA DAO or in either STAA or LTAA DASD. There were no associations of cocaine or methamphetamine use measures with subcortical volume measures in either STAA or LTAA DASD. Within the combined alcoholic groups, there were no associations of any subcortical volume measures with nicotine use measures (Wilks' λ_7,75_ = 0.91, p > .41). There were no associations of days abstinent from alcohol or any drug within any group. There were no associations of subcortical volumes with lifetime or current total psychiatric, mood, anxiety, or internalizing diagnoses.

### 3.4 Comparing DAO vs. DASD on cognitive measures

Comparing DAO and DASD (combined STAA and LTAA) on the cognitive measures that showed impairment in STAA vs. NSAC, the multivariate test was not significant (Wilks' λ_9,154_ = 0.949, p > 0.51). The only significant correlations of subcortical volumes with CANTAB tests were negative associations in STAA DAO of accumbens and putamen volumes with that were of the accumbens and putamen with reaction time to targets on the Affective Go/No-go Task (r = − .341 and − .330, both p's < 0.05, uncorrected for multiple comparisons).

## 4 Discussion

There are three major and three minor findings in this paper. First, we found smaller subcortical volumes for the accumbens, hippocampus, and pallidum in STAA DAO vs. NSAC. Second, we found smaller subcortical volumes of the same structures (plus the putamen) in LTAA DAO vs. NSAC, with the size of the differences from NSAC being consistently (but not significantly) smaller for LTAA compared to STAA. Third, we did not observe any differences from NSAC for either STAA DASD or LTAA DASD. For STAA, DASD evidenced larger volumes than DAO for the accumbens, hippocampus, pallidum, putamen and thalamus. Within LTAA, there were no significant differences in subcortical volumes between those DAO and those DASD. Fourth, within STAA DAO, subcortical volumes were moderately negatively correlated with alcohol use, but no associations with cocaine, methamphetamine, or nicotine use measures nor with days abstinent on alcohol, stimulants or nicotine was evident in any group. Fifth, alcoholics DAO and DASD (combined STAA and LTAA) did not differ on any cognitive measures that showed impairment in STAA vs. NSAC. Finally, in LTAA, individuals with a cocaine disorder had lower volumes of the accumbens, hippocampus and thalamus than those with a methamphetamine disorder, suggesting that the inflammation secondary to a cocaine disorder may resolve with long-term abstinence while that due to a methamphetamine disorder does not.

### 4.1 Subcortical volumes are reduced in STAA DAO compared to NSAC

The smaller volumes were found in the accumbens, hippocampus, and pallidum. The largest effect was for the hippocampus, consistent with the literature on the morbid effects of chronic alcohol abuse on hippocampal volumes and function (Agartz et al., 1999; Bonthius et al., 2001; Harding et al., 1997; Sullivan et al., 1995, 1996). The findings for the other regions are consistent with prior research showing smaller volumes of reward-related subcortical structures in alcoholics compared to controls (Makris et al., 2008; Oscar-Berman and Song, 2011).

### 4.2 Subcortical volumes are reduced in LTAA DAO compared to NSAC

In our earlier study of LTAA recruited from the San Francisco Bay Area, we did not find a group (LTAA vs. NSAC) main effect for subcortical volume. Our finding of such an effect (lower volumes in LTAA vs. NSAC for the accumbens, hippocampus, pallidum, putamen and thalamus) in the current study is likely due to a combination of both sample differences and imaging protocol improvements in Oahu vs. California. The current (Oahu) sample did not differ in age from the California sample, but started drinking on average 1.5 years earlier (14.4 ± 4.0 vs. 15.8 ± 4.6 yrs; F_1,155_ = 5.0, p = 0.026, es = 3.1). They also had much less education (13.6 ± 2.3 vs. 15.5 ± 2.1 yrs; F_1,155_ = 25.0, p < 0.0001, es = 13.9), much higher body mass indices (29.3 ± 5.4 vs. 25.9 ± 3.8; F_1,155_ = 17.5, p < 0.0001, es = 10.1), and a trend toward greater alcohol doses (199.9 ± 156 vs. 157.3 ± 122 drinks/mo; F_1,155_ = 3.2, p = 0.078, es = 2.0). The imaging studies on Oahu were carried out on a 3.0T magnet vs. a 1.5T magnet in California, with 5 year newer software and hardware in Oahu. LTAA in Oahu were not as healthy as those in California (much higher BMI) and were much less highly educated than those in California, possibly indicating less brain functional reserve capacity — both of these factors may have limited their capacity for recovery from the brain morbidity of alcoholism (thus evidencing lower than NSAC subcortical volumes even after multi-year abstinence). Additionally, their greater alcohol use may have given them a greater alcohol brain morbidity burden to recover from. Finally, the improved imaging protocol may have been more sensitive in measuring the effects of alcoholism on subcortical structures. We believe the major import of this result is that one cannot generalize from one study of LTAA to the population of LTAA. LTAA cohorts may differ in alcoholism severity and in their ability to recover from such burden. Finally, given the cross-sectional nature of this study, we cannot definitively state that differences between STAA and LTAA are the result of differences in abstinence durations — the differences may be due to some unmeasured cohort differences. Nonetheless, the similarity of STAA and LTAA on demographic and alcohol use variables supports the idea that differences between STAA and LTAA are a consequence of differences in length of abstinence.

It is important to note that the differences in subcortical volumes in LTAA DAO vs. NSAC were numerically (but not statistically) smaller than the differences between STAA DAO and NSAC. The (numerically) smaller differences are consistent with either partial recovery of subcortical volumes with long-term abstinence or with selective survivorship (i.e., STAA with less subcortical volume loss are more likely to survive to long-term abstinence). These alternative hypotheses can only be decided between with an adequately powered longitudinal study. The lack of a statistically significant difference between subcortical volumes in STAA DAO and LTAA DAO is consistent either with no recovery of subcortical volumes with long-term abstinence or with inadequate power of the test of differences. Once again, this speaks to the need for adequately powered longitudinal studies.

### 4.3 The effect of comorbid stimulant abuse/dependence on subcortical volumes in STAA and LTAA

STAA or LTAA with comorbid stimulant dependence had subcortical volumes which did not differ from NSAC. In STAA, DASD showed larger subcortical volumes than DAO; in LTAA, no such differences were evident, yet the lower volumes in LTAA DAO vs. NSAC were no longer present in LTAA DASD. This set of results is consistent with either of the two hypotheses. In the first hypothesis, comorbid stimulant abuse/dependence protects individuals from alcohol dependence induced atrophy. In the second hypothesis, comorbid stimulant abuse/dependence causes inflammation of the already atrophied tissue, masking the effects of alcohol dependence induced subcortical atrophy, with the inflammatory effect diminishing with long-term abstinence. Our cognitive data is highly supportive of the inflammation hypothesis. If stimulants were protective, we would have expected alcoholics DASD to show less cognitive impairments than alcoholics DAO – the results showed DASD evidenced numerically (but not statistically) more impairment than DAO. The lack of associations of subcortical volumes with cognitive performance measures in STAA DAO is consistent with subjects DAO having reached a threshold of subcortical volume reduction that negatively impacts cognitive function.

### 4.4 Limitations of the current study

There are four major limitations of this study: First, it is observational rather than experimental. We can't be sure that the differences between samples DAO and DASD have to do with the abuse of stimulants rather than some unmeasured cohort differences between the samples. Experimental studies of addiction (where subjects are randomly assigned to alcohol only or alcohol and stimulants) are only possible with animals, which have their own inherent limitations, especially in studies of abstinence (where the human condition necessarily includes issues of choice, motivation, and compliance). Nonetheless, the demographic similarity between alcoholics DAO and DASD suggests that the differences between groups are likely the result of stimulant abuse. Second, the study is cross-sectional rather than longitudinal, which limits our ability to definitively attribute differences between STAA and LTAA to duration of abstinence. This limitation was discussed above. Third, STAA were abstinent a minimum of six weeks. The study is therefore silent with regard to subcortical volume effects of alcohol and stimulant abuse and dependence in very early abstinence. This might be particularly relevant to our lack of finding any associations of subcortical volumes with days abstinent in STAA. Such effects might be present in the first days and weeks of abstinence. Finally, we acknowledge that we did not actually measure inflammation, but inferred it from the literature, the lack of findings on smaller subcortical volumes in the alcoholic samples DASD, and the comparable or greater severity of functional impairments in alcoholics DASD vs. DAO. Inflammation and atrophy could be more directly inferred from MR spectroscopy studies which could measure subcortical neuronal markers (NAA) and metabolites that reflect inflammation.

## Figures and Tables

**Table 1:** Demographic and substance use measures.

	STAA				LTAA				NSAC	
	DAO N = 36		DASD N = 41		DAO N = 47		DASD N = 43		N = 74	
	♂ N = 24	♀ N = 12	♂ N = 24	♀ N = 17	♂ N = 28	♀ N = 19	♂ N = 19	♀ N = 24	♂ N = 37	♀ N = 37
*Demographics*										
Age (yrs)	49 ± 7	50 ± 7	44 ± 6	43 ± 4	48 ± 7	50 ± 5	49 ± 8	49 ± 7	47 ± 7	49 ± 8
Years of education	14 ± 3	14 ± 3	13 ± 2	13 ± 2	14 ± 2	13 ± 2	14 ± 3	13 ± 2	16 ± 3	16 ± 3
Prop of 1st degree relative problem drinkers	.27 ± .28	.45 ± .34	.26 ± .26	.41 ± .30	.28 ± .29	.40 ± .27	.23 ± .29	.41 ± .33	.13 ± .19	.23 ± .23
Prop of 1st degree relative problem drug users	.12 ± .17	.12 ± .18	.22 ± .25	.34 ± .30	.14 ± .27	.18 ± .19	.16 ± .28	.31 ± .30	.03 ± .09	.05 ± .13

*Alcohol use variables*										
Duration of alcohol use (mo)	353 ± 110	356 ± 95	333 ± 80	274 ± 66	299 ± 114	297 ± 111	293 ± 99	297 ± 118	252 ± 143	270 ± 145
Average alcohol dose (drinks/mo)	245 ± 265	109 ± 76	201 ± 150	165 ± 122	230 ± 185	215 ± 183	214 ± 154	154 ± 111	10 ± 9	8 ± 8
Duration of peak use (mo)	133 ± 111	92 ± 89	90 ± 89	56 ± 36	84 ± 76	83 ± 58	124 ± 111	118 ± 99	95 ± 67	84 ± 79
Peak dose (drinks/mo)	473 ± 552	228 ± 153	433 ± 229	377 ± 254	379 ± 293	377 ± 265	368 ± 258	293 ± 341	18 ± 15	17 ± 16
Abstinence duration (yrs)	67 ± 18	73 ± 20	75 ± 19	76 ± 23	2638 ± 2761	2847 ± 2587	2546 ± 2116	2997 ± 2416	N/A	N/A

*Stimulant use variables*										
Cocaine lifetime dose (g/mo)	.03 ± .13	.19 ± .64	22 ± 45	5 ± 8	.4 ± 2.1	0	54 ± 111	21 ± 24	0	0
Cocaine peak dose (g/mo)	.04 ± .2	.19 ± .64	38 ± 92	10 ± 13	.4 ± 2.1	0	72 ± 151	27 ± 28	0	0
Methamphetamine Lifetime Dose (g/mo)	0	0	20 ± 35	29 ± 63	0	0	14 ± 21	14 ± 17	0	0
Methamphetamine peak dose (g/mo)	0	0	24 ± 56	58 ± 168	0	0	17 ± 24	18 ± 20	0	0

*Smoking variables*										
Lifetime nicotine dependence # (%)	89	84	70	71	74	63	100	76	11	16
Current nicotine dependence # (%)	90	89	43	47	30	53	42	57	0	5


	Effect Size (partial η^2^)						
	STAA DAO vs NSAC	STAA DASD vs NSAC	STAA DAO vs STAA DASD	LTAA DAO vs. NSAC	LTAA DASD vs. NSAC	LTAA DAO vs. LTAA DASD	(STAA vs. LTAA) DAO
*Demographics*							
Age (yrs)	0.3	11.9⁎⁎⁎	17.8⁎⁎⁎	0.2	0	0.2	0
Years of education	9.4⁎⁎	22.6⁎⁎⁎	4.8	18.3⁎⁎⁎	15.4⁎⁎⁎	0	1.3
Prop of 1st degree relative problem drinkers	6.9⁎⁎	13.5⁎⁎⁎	2	11.4⁎⁎⁎	11.5⁎⁎⁎	0	0.1
Prop of 1st degree relative problem drug users	3.6	28.1⁎⁎⁎	13.3⁎⁎⁎	8.3⁎⁎	15.2⁎⁎⁎	2.2	0.9

*Alcohol use variables*							
Duration of alcohol use (mo)	9.8^a^	2.8^a^	6.1⁎	1.9^a^	1.6^a^	0	5.8⁎
Average alcohol dose (drinks/mo)	26.1^a^	49.4^a^	0.2	44.2^a^	52.5^a^	1.6	1.2
Duration of peak use (mo)	1.5^a^	1.3^a^	5.9^a^	0.2^a^	3.0^a^	4.3	2.5
Peak dose (drinks/mo)	24.3^a^	62^a^	0	49^a^	40.2^a^	0.7	0.1
Abstinence duration (yrs)	89.7^a^	89.3^a^	2.4^a^	38^a^	48.6^a^	0	28.6^a^

*Stimulant use variables*							
Cocaine lifetime dose (g/mo)	0.7	8.7^a^	7.5^a^	0.9	12.6^a^	10.3^a^	0.1
Cocaine peak dose (g/mo)	0.9	6.7^a^	5.9^a^	0.9	12.4^a^	10.2^a^	0.1
Methamphetamine lifetime dose (g/mo)	0.5	13.7^a^	10.3^a^	0.6	26.6^a^	24.2^a^	0
Methamphetamine peak dose (g/mo)	0.5	7.3^a^	4.9^a^	0.6	27.1^a^	24.7^a^	0

*Smoking variables*							
Lifetime nicotine dependence # (proportion)	4.3⁎	11.3⁎⁎⁎	1.6	11.9⁎⁎⁎	20.7⁎⁎⁎	1.1	1.6
Current nicotine dependence # (proportion)	10.7⁎⁎⁎	11.7⁎⁎⁎	0	11.1⁎⁎⁎	14.2⁎⁎⁎	0.4	0

LTAA, Long-Term Abstinent Alcoholics; NSAC, Nonsubstance Abusing Controls; STAA, Short-Term Abstinent Alcoholics; DAO, Dependent On Alcohol Only; DASD, dependent On Alcohol With A Stimulant Disorder (abuse or dependence). Effect is significant:

⁎ p ≤ .05.

⁎⁎ p ≤ .01.

⁎⁎⁎ p ≤ .001.

a Statistical comparisons are invalid since this variable is related to the inclusion criteria.

**Table 2:** Subcortical volumes (mm^3^) (Mean ± S.D.) (adjusted for inter-subject variability in intracranial vault volume).

	STAA				LTAA				NSAC	
	DAO N = 36		DASD N = 41		DAO N = 47		DASD N = 43		N = 74	
	♂ N = 24	♀ N = 12	♂ N = 24	♀ N = 17	♂ N = 28	♀ N = 19	♂ N = 19	♀ N = 24	♂ N = 37	♀ N = 37
Accumbens	1910 ± 451	1802 ± 385	2036 ± 261	2138 ± 344	1985 ± 257	1906 ± 453	1994 ± 206	2022 ± 267	2049 ± 289	2065 ± 195
Amygdala	4943 ± 320	4816 ± 403	5117 ± 361	5027 ± 412	4886 ± 421	4770 ± 1087	5061 ± 346	5005 ± 318	4982 ± 353	4921 ± 306
Caudate	8383 ± 1732	8014 ± 1348	9032 ± 1243	9087 ± 1403	8780 ± 1702	8562 ± 1495	9009 ± 1450	8637 ± 1422	8550 ± 1555	8692 ± 1437
Hippocampus	12010 ± 1206	11778 ± 1035	12600 ± 723	12753 ± 911	12197 ± 1343	11880 ± 2565	12562 ± 797	12583 ± 939	12706 ± 884	12651 ± 633
Pallidum	6228 ± 643	6183 ± 783	6443 ± 369	6416 ± 552	6399 ± 266	6210 ± 1150	6466 ± 311	6619 ± 805	6480 ± 363	6638 ± 564
Putamen	17050 ± 103	16946 ± 981	17149 ± 919	17105 ± 675	17309 ± 834	15955 ± 3284	17331 ± 613	17328 ± 622	17294 ± 786	17201 ± 700
Thalamus	24938 ± 801	24977 ± 1033	25409 ± 514	25323 ± 724	24948 ± 787	24043 ± 4453	25186 ± 632	25180 ± 654	24247 ± 795	25304 ± 561

LTAA, Long-Term Abstinent Alcoholics; NSAC, NonSubstance Abusing Controls; STAA, Short-Term Abstinent Alcoholics; DAO, Dependent on Alcohol Only; DASD, Dependent on Alcohol with a Stimulant Disorder (abuse or dependence).
